# Clinical features of persistent postural-perceptual dizziness coexisting with Meniere’s disease in comparison with Meniere’s disease alone

**DOI:** 10.3389/fneur.2024.1425647

**Published:** 2024-07-31

**Authors:** Chihiro Yagi, Akira Kimura, Keito Ishida, Takeshi Takahashi, Ryota Kai, Tatsuya Yamagishi, Shinsuke Oshima, Shuji Izumi, Arata Horii

**Affiliations:** Department of Otolaryngology, Head and Neck Surgery, Niigata University Graduate School of Medical and Dental Sciences, Niigata, Japan

**Keywords:** persistent postural perceptual dizziness, Meniere’s disease, Niigata PPPD questionnaire (NPQ), dizziness handicap inventory (DHI), hospital anxiety and depression scale (HADS)

## Abstract

**Background:**

Persistent postural-perceptual dizziness (PPPD) is a chronic vestibular syndrome often triggered by acute or episodic vestibular syndromes, such as Meniere’s disease (MD). According to the diagnostic criteria, PPPD may coexist with other structural diseases, and the evidence of another active illness does not necessarily exclude PPPD diagnosis. However, persistent symptoms, even those meeting the PPPD criteria even long after Meniere’s attack, are often overlooked as potential PPPD precipitated by MD. Some clinicians overlook PPPD in such patients, treating them solely for MD once diagnosed. Since a treatment strategy for PPPD is completely different from that for MD, this may result in the deprivation of adequate treatments.

**Objectives:**

To emphasize the importance of diagnosing PPPD coexisting with MD including not treating such patients solely for MD, and to compare the clinical features of PPPD and MD.

**Methods:**

Vestibular function tests, including canal paresis (CP)%, c- and o-vestibular myogenic potentials, vestibulo-ocular reflex-direction preponderance, and posturography and clinical symptom scales, including the Dizziness Handicap Inventory, Niigata PPPD Questionnaire, and Hospital Anxiety and Depression Scale, were compared between 105 PPPD patients with MD or other precipitants and 130 patients with MD alone. The clinical symptom scales were further compared between 23 patients with PPPD coexisting with MD and those with MD alone.

**Results:**

The CP% was significantly higher in patients with MD than in those with PPPD. However, the total and subscores of all three clinical symptom scales were higher in patients with PPPD than in those with MD. The total score on all clinical symptom scales was higher in patients with PPPD coexisting with MD than in those with MD alone.

**Conclusion:**

Persistent postural-perceptual dizziness development from a precipitating MD may be associated with more severe clinical symptoms. Thus, clinical symptom scales may be useful for detecting PPPD in patients with Meniere’s disease.

## 1 Introduction

Vestibular syndromes are classified into three categories based on the disease onset: acute, episodic, and chronic according to the International Classification of Diseases 11th revision (ICD-11) of the World Health Organization (WHO) (1). Among these categories, persistent postural-perceptual dizziness (PPPD) is the most frequent cause of the chronic vestibular syndrome that usually follows acute or episodic vestibular diseases such as vestibular neuritis and Meniere’s disease, respectively. Precipitating diseases of PPPD are usually resolved; however, episodic diseases may be superimposed on PPPD. Indeed, PPPD may coexist with other diseases or disorders and evidence of another active illness does not necessarily exclude PPPD diagnosis according to notes to the diagnostic criteria of PPPD (2). Meniere’s disease, a representative of the episodic vestibular syndrome, usually demonstrates none or few vestibular symptoms during intermittent periods after fully recovered from vertigo spells. Therefore, vestibular symptoms during intermittent periods of Meniere’s attacks should not be considered Meniere’s symptoms but a possible development of PPPD. The other diagnostic dilemma is that unilateral peripheral vestibulopathy due to Meniere’s disease is usually compensated for over time; however, it might demonstrate PPPD-like symptoms in limited patients. Even in such a case, PPPD coexisting with Meniere’s disease can be sorted from Meniere’s disease alone by visual sensitivities and characteristics of exacerbated symptoms, which is long-lasting once evoked. Taking careful histories in combination with several questionnaires may help to point a development of PPPD from Meniere’s disease.

Nonetheless, in clinical settings, there is often confusion and misunderstanding about persistent symptoms meeting with PPPD criteria after Meniere’s attack, which are frequently overlooked as potential PPPD precipitated by Meniere’s disease. Some clinicians continue to treat such PPPD patients solely for Meniere’s disease, overlooking the potential for concurrent PPPD. Since the treatment strategy for PPPD completely differs from that for Meniere’s disease, this oversight may deprive patients of adequate treatments.

In this study, we aimed to highlight the importance of diagnosing PPPD coexisting with Meniere’s disease, including not treating such patients solely for Meniere’s disease. Demographic data, vestibular function tests, and answers to several clinical symptom scales, including the Dizziness Handicap Inventory (DHI) for everyday handicaps due to dizziness, the Niigata PPPD Questionnaire (NPQ) for symptom exacerbations in PPPD, and the Hospital Anxiety and Depression Scale (HADS) for mental conditions, were compared between patients with PPPD and Meniere’s disease. Additionally, comparisons were made between PPPD coexisting with Meniere’s disease and Meniere’s disease alone. These comparisons reveal significant differences between the conditions and suggest the importance of adding PPPD diagnosis to patients with Meniere’s disease who exhibit persistent symptoms.

## 2 Materials and methods

This study was conducted in accordance with the Declaration of Helsinki and was approved by the IRB of Niigata University Hospital. Vestibular function tests and clinical symptom scales (DHI, NPQ, and HADS) were performed at non-acute stage within approximately 1 month of the first visit to the hospital before starting treatment.

### 2.1 Patients and diagnosis

Consecutive patients diagnosed with Meniere’s disease or PPPD between October 2019 and August 2023 at the Department of Otolaryngology, Head and Neck Surgery of Niigata University Hospital were enrolled in the study. PPPD or Meniere’s disease was diagnosed using the Barany Society criteria (2, 3). All patients provided informed consent. There were total of 130 and 105 patients with Meniere’s disease and PPPD, respectively. Precipitating conditions of PPPD are shown in Table 1. Of the 105 patients with PPPD, 12 exhibited comorbid Meniere’s disease. Data of these 12 patients and 11 PPPD patients with MD diagnosed from May 2015 and September 2019 were analyzed in combination as PPPD coexisting with MD.

**Table 1:** Precipitating conditions of patients with persistent postural-perceptual dizziness (PPPD) (*n* = 105).

Acute attack of peripheral vestibular vertigo	*n* = 39
BPPV	*n* = 17
Meniere’s disease	*n* = 12
No specific precipitants	*n* = 8
Sudden deafness with vertigo	*n* = 6
Chronic anxiety disorders	*n* = 5
Vestibular neuritis	*n* = 5
Phobia	*n* = 4
Post-traumatic inner ear concussion	*n* = 3
Orthostatic dysregulation	*n* = 2
Panic attack	*n* = 2
Cerebellar infarction	*n* = 1
Ramsay Hunt syndrome	*n* = 1

BPPV, Benign paroxysmal positional vertigo.

Regarding the judgment of Meniere’s disease alone or PPPD coexisting with Meniere’s disease, we followed the following principle: patients with Meniere’s disease alone usually demonstrate few or no symptoms during the intermittent period of vertigo spells. However, the characteristics of vestibular symptoms with regard to their persistence should be carefully examined if patients complain of vestibular symptoms that are not associated with vertigo spells. Moment flare-ups evoked by motion/visual stimulation would suggest unilateral vestibular hypofunction due to Meniere’s disease alone, whereas the existence of persistent symptoms without motion would suggest that PPPD coexists with Meniere’s disease.

### 2.2 Clinical symptom scale

#### 2.2.1 Dizziness handicap inventory

The DHI is a standard 25-question questionnaire designed to quantitatively evaluate the handicap degree felt by patients with vestibular disorders in their daily lives (4, 5). The total score ranged from 0 to 100, with 0 and 100 indicating no disability and severe disability, respectively.

#### 2.2.2 Niigata PPPD questionnaire

The NPQ is a self-administered questionnaire used to screen and assess PPPD severity (6). The NPQ consists of 12 questions that assess the degree of symptom exacerbation for three exacerbating factors: upright posture or walking, active or passive motion, and visual stimulation. The severity of each factor was evaluated using four questions, with scores ranging from 0 (none) to 6 (unbearable). Thus, the total score for each factor was 24 and the full score for the NPQ was 72, with higher scores indicating greater severity.

#### 2.2.3 Hospital anxiety and depression scale

The HADS consists of self-administered anxiety and depression subscales. Each HADS subscale was assessed using seven questions (7). Each question was scored on a scale of 0 (not at all) to 3 (most of the time, very often). Therefore, the total score for each HADS subscale was 21 and the full HADS score was 42, with higher scores indicating higher anxiety and depression levels.

### 2.3 Vestibular function test

#### 2.3.1 Bithermal air caloric testing

Air at 26°C and 45°C each for 60 s was used to perform bithermal caloric testing. Each external auditory canal was stimulated two times with a 5-min interval between the stimulations. Electronystagmography was used to measure the maximum slow-phase velocity, and Jongkee’s index formula (8) was used to calculate canal paresis (CP). A CP value >20% was considered to indicate significant unilateral caloric weakness.

#### 2.3.2 Cervical and ocular vestibular-evoked myogenic potential

The Neuropack® system (Nihon Kohden, Japan) was used to determine the cervical and ocular vestibular-evoked myogenic potentials (cVEMP and oVEMP) to assess the saccular and utricular functions, respectively. Clicks (0.1-ms rarefactive square waves of 105-dB nHL) were used to induce cVEMP. We recorded the oVEMP by generating 500-Hz tone bursts (4-ms plateau and 1-ms rise and fall) by a hand-held electromechanical vibrator (Minishaker®, Bruel & Kjaer, Denmark), which was used as bone-conducted stimuli. Amplitudes and latencies were measured at response peaks, which occurred at approximately 13 and 23 ms for cVEMP and 10 and 15 ms for oVEMP, respectively, depending on the stimulus. The peak-to-peak (PP) amplitudes were obtained using the differences between the peak amplitudes. The asymmetry ratio (AR) was calculated using the formula to compare the two ears: AR = (right − left)/(right + left) × 100 (%) in the raw PP amplitude (9). An AR >33.3% was defined as unilateral saccular (cVEMP) or utricular (oVEMP) dysfunction.

#### 2.3.3 Rotatory chair test

The Nistamo21 IRN 2 (Morita, Japan) was used to perform the rotatory chair test. The patients sat in a rotatory chair to which a pendulum-like rotation was applied so that the maximum head angular velocity was 50°/s at a stimulation frequency of 0.1 Hz. The angular velocity of the eye movements was monitored and analyzed. We calculated the vestibulo-ocular reflex directional preponderance, and a value >12% was considered significant.

#### 2.3.4 Posturography

The patients underwent static posturography on a solid surface using Gravicoda® (ANIMA Corp., Japan) with open and closed eyes. The elliptical balance area (cm^2^) tested on a solid surface was used as a representative index of the postural sway.

### 2.4 Statistical analyses

Comparisons were conducted between patients with PPPD (*n* = 105) and those with Meniere’s disease alone (*n* = 130), as well as between patients with PPPD coexisting with Meniere’s disease (*n* = 23) and those with Meniere’s disease alone. Between the groups, age and sex were tested using the Mann–Whitney U test and Pearson’s chi-square test, respectively. The Mann–Whitney U test was used to test all clinical symptom scales, including the DHI, NPQ, and HADS, as well as the vestibular function tests, between the groups. *p* values <0.05 were considered as significant.

## 3 Results

### 3.1 Comparison between PPPD and Meniere’s disease

Table 2 shows the demographic data and clinical symptom scales of PPPD and Meniere’s disease. No age difference was observed between patients with PPPD and those with Meniere’s disease. Pearson’s chi-square test revealed that PPPD was significantly more female-dominant than Meniere’s disease (*p* < 0.05).

**Table 2:** Demographic data and clinical symptom scales of PPPD and Meniere’s disease.

	PPPD (*n* = 105) (mean ± SD)	Meniere’s disease (*n* = 130) (mean ± SD)	*p* value
Age	49.9 ± 16.0	52.2 ± 14.5	0.1683
Men/Women	26/79	51/79	<0.05
DHI			
Total	56.7 ± 18.5	34.6 ± 24.0	<0.0001
Physical	16.1 ± 6.2	8.9 ± 6.9	<0.0001
Emotional	20.1 ± 7.4	13.6 ± 9.5	<0.0001
Functional	20.6 ± 8.6	12.5 ± 9.9	<0.0001
NPQ			
Total	41.8 ± 15.2	23.7 ± 20	<0.0001
Upright/walking	13.2 ± 5.4	7.3 ± 7.0	<0.0001
Motion	14.1 ± 5.1	8.7 ± 6.7	<0.0001
Visual	14.5 ± 6.5	7.6 ± 7.1	<0.0001
HADS			
Total	18.5 ± 8.0	13.6 ± 7.3	<0.0001
Anxiety	9.5 ± 4.5	6.8 ± 4.2	<0.0001
Depression	9.0 ± 4.6	6.9 ± 3.9	<0.001

Regarding the clinical symptom scales (Table 2), the total DHI score and the physical, emotional, and functional sub-scores of the DHI were all significantly higher in patients with PPPD than in those with Meniere’s disease (*p* < 0.0001). The total scores of the NPQ, upright/walking, motion, and visual sub-scores of the NPQ were significantly higher in patients with PPPD than in those with Meniere’s disease (*p* < 0.0001). The total HADS score, anxiety, and depression sub-scores of the HADS were significantly higher in patients with PPPD than in those with Meniere’s disease (*p* < 0.001 ~ 0.0001).

Table 3 demonstrates the results of the vestibular function test of PPPD and Meniere’s disease. The CP% of Meniere’s disease was 22.4 ± 20.1 [mean ± standard deviation (SD)], which was significantly higher than that of PPPD (16.9 ± 18.8) (*p* < 0.05). The asymmetry ratio of cVEMP and oVEMP were not different between PPPD and Meniere’s disease, and all exhibited normal results. The DP% of VOR in the rotatory chair test was not different between PPPD and Meniere’s disease. The differences in the ellipse area of postural sway in both eyes open/closed conditions between PPPD and Meniere’s disease were insignificant.

**Table 3:** Vestibular function test of PPPD and Meniere’s disease.

	PPPD (*n* = 105) (mean ± SD)	Meniere’s disease (*n* = 130) (mean ± SD)	*p* value
CP (%)	16.9 ± 18.8	22.4 ± 20.1	<0.05
Asymmetry ratio of cVEMP (%)	16.2 ± 11.5	19.2 ± 21.5	0.9221
Asymmetry ratio of oVEMP (%)	16.5 ± 18.1	19.4 ± 22.2	0.4735
VOR-DP (%)	7.2 ± 6.9	10.4 ± 14.1	0.1023
Ellipse area (eyes open) (cm^2^)	7.0 ± 7.8	5.0 ± 2.7	0.4229
Ellipse area (eyes closed) (cm^2^)	9.9 ± 9.4	8.0 ± 8.2	0.2053

### 3.2 Comparing the clinical symptom scales between PPPD coexisting with Meniere’s disease and Meniere’s disease alone

**Figure 1:**
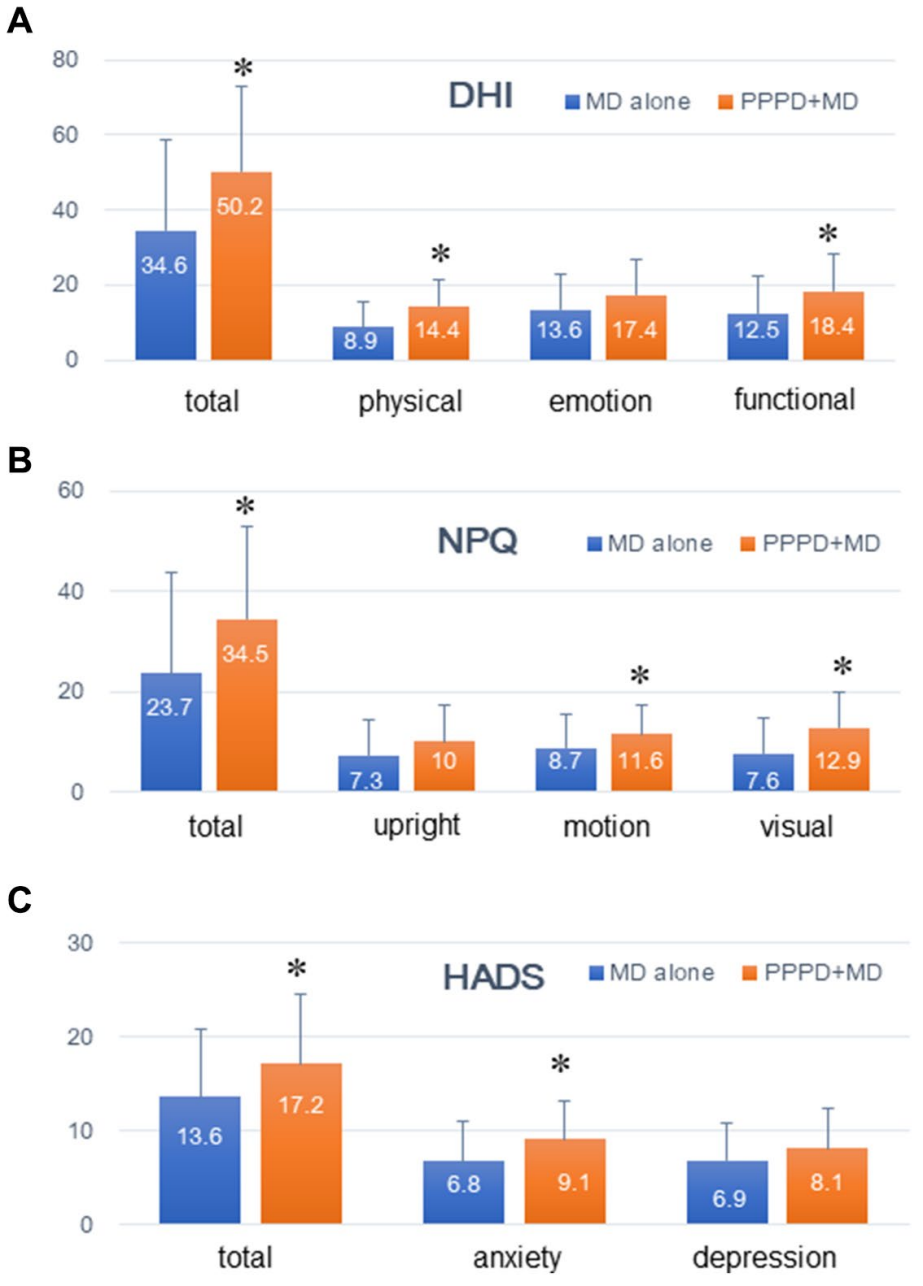
Comparing the clinical symptom scales between PPPD coexisting with Meniere’s disease and Meniere’s disease alone. The total and sub-scores of the Dizziness Handicap Inventory (DHI) except for DHI-E (emotion), were all higher in patients with PPPD coexisting with Meniere’s disease than in those with Meniere’s disease alone. The total score and all sub-scores of the Niigata PPPD Questionnaire except for the upright/walking subscore, were higher in patients with PPPD coexisting with Meniere’s disease than in those with Meniere’s disease alone. The total and anxiety sub-scores of the Hospital Anxiety and Depression Scale were higher in patients with PPPD coexisting with Meniere’s disease than in those with Meniere’s disease alone.

Figure 1 illustrates the clinical symptom scales (DHI, NPQ, and HADS) of patients with Meniere’s disease alone (*n* = 130) and those with PPPD coexisting with Meniere’s disease (*n* = 23). Data are represented as mean ± SD, and the numeral in the column is a mean value. The total and sub-scores of DHI, except for DHI-E (emotion), were significantly higher in patients with PPPD coexisting with Meniere’s disease than in those with Meniere’s disease alone (*p* < 0.05). The total score and all sub-scores of NPQ, except for the upright/walking sub-score, were significantly higher in patients with PPPD coexisting with Meniere’s disease than in those with Meniere’s disease alone (*p* < 0.05). The total and anxiety sub-score of the HADS were significantly higher in patients with PPPD coexisting with Meniere’s disease than in those with Meniere’s disease alone (*p* < 0.05).

## 4 Discussion

Herein, the CP% of PPPD (16.9%) was within the normal range (< 20%), whereas that of Meniere’s disease (22.4%) was significantly higher than that of PPPD. This suggests a slightly impaired horizontal canal function in Meniere’s disease (Table 3). Similarly, the asymmetry ratio of c- and o-VEMPs tended to be higher in Meniere’s disease than in PPPD (Table 3). Thus, the vestibular function may be slightly suppressed in Meniere’s disease compared to PPPD, in which all vestibular function parameters were within the normal range (Table 3). Normal vestibular function in PPPD was consistent with the PPPD concept, that is, persistent vestibular symptoms after complete recovery from precipitating conditions (Table 1). Contrastingly, slightly impaired vestibular function was observed in Meniere’s disease. However, all clinical symptom scales, including DHI, NPQ, and HADS, were higher in PPPD than in Meniere’s disease, suggesting an overall more severe subjective disturbance in PPPD (Table 2).

The higher DHI scores in PPPD than in Meniere’s disease were consistent with those reported in previous studies (10). The total DHI score for PPPD was 56.7, which exceeded the upper limit of moderate handicap reaching the severe level, whereas that of Meniere’s disease (34.6) was approximately the upper limit of mild handicap (Table 2). The cutoff point for the total NPQ score developed to detect characteristic symptom exacerbation in PPPD was 27 (6). Therefore, the total NPQ score for Meniere’s disease (23.7) was within the normal range, whereas that of PPPD was 41.8. This exceeded the cut-off value, which was significantly higher than that of Meniere’s disease. Symptom exacerbation by motion/visual stimulation could be seen not only in PPPD but also in unilateral vestibular diseases, including Meniere’s disease; however, the above results suggest that symptom exacerbation is more characteristic of PPPD than Meniere’s disease. The anxiety and depression sub-scores of PPPD and Meniere’s disease were within the normal range (<11) (Table 2), although both diseases could have anxiety as a mental background (2, 11). However, the total score and anxiety/depression sub-scores of the HADS were higher in patients with PPPD than in those with Meniere’s disease (Table 2). These findings suggest that the overall subjective symptoms of PPPD as measured by DHI, NPQ, and HADS are more severe than those of Meniere’s disease. Thus, PPPD should be distinguished from Meniere’s disease, which should in turn receive adequate treatments as for PPPD.

Clinical symptom scales were compared between PPPD coexisting with Meniere’s disease and Meniere’s disease alone, considering that possibilities remained that the above-mentioned differences between PPPD and Meniere’s disease might be due to other factors such as precipitants and PPPD comorbidities. Most of the clinical symptom scales, except for DHI-E (emotional), NPQ-upright/walking, and HADS-D (depression), were higher in PPPD coexisting with Meniere’s disease than in Meniere’s disease alone (Figure 1). These results suggest that PPPD development from precipitating Meniere’s disease may be associated with the development of more severe symptoms, which may be accompanied by a drastic change in pathophysiology from Meniere’s disease to PPPD. The most frequent precipitating diseases of PPPD are acute or episodic vestibular syndromes such as BPPV, Meniere’s disease, and vestibular neuritis, with psychological disorders also being substantial contributors (2). The actual percentage of PPPD development from Meniere’s disease is uncertain, whereas BPPV, a representative episodic vestibular syndrome, develops PPPD less frequently than vestibular neuritis (12). Clinicians who follow Meniere’s disease should not overlook PPPD development from Meniere’s disease, given that the clinical symptoms of PPPD are more severe than those of Meniere’s disease (Table 2). Therefore, anamnesis of persistent vestibular symptoms that are not associated with vertigo spells is crucial. Additionally, clinical symptom scales such as the DHI, NPQ, and HADS may be useful for distinguishing PPPD development from Meniere’s disease.

This study has a limitation in that it was a cross-sectional study comparing several items between PPPD and Meniere’s disease. Future research should include a prospective cohort study to explore PPPD development from Meniere’s disease.

In conclusion, PPPD demonstrated normal vestibular function, whereas Meniere’s disease exhibited slightly suppressed horizontal canal function. However, all clinical symptom scales, such as the DHII, NPQ, and HADS, were even higher in patients with PPPD than in those with Meniere’s disease. Most of these scales were also higher in patients with PPPD coexisting with Meniere’s disease than in those with Meniere’s disease alone. These findings indicate that the overall subjective symptoms of patients with PPPD may be more severe than those of Meniere’s disease, emphasizing the need for distinction from Meniere’s disease, which should in turn receive adequate treatment as for PPPD.

## Data availability statement

The raw data supporting the conclusions of this article will be made available by the authors, without undue reservation.

## Ethics statement

The studies involving humans were approved by Ethics Committee of Niigata University. The studies were conducted in accordance with the local legislation and institutional requirements. The participants provided their written informed consent to participate in this study.

## Author contributions

CY: Conceptualization, Data curation, Writing – review & editing. AK: Data curation, Formal analysis, Writing – review & editing. KI: Data curation, Writing – review & editing, Formal analysis. TT: Conceptualization, Data curation, Writing – review & editing. RK: Conceptualization, Data curation, Writing – review & editing. TY: Conceptualization, Data curation, Writing – review & editing. SO: Conceptualization, Data curation, Writing – review & editing. SI: Conceptualization, Data curation, Writing – review & editing. AH: Writing – original draft, Writing – review & editing, Conceptualization, Data curation, Funding acquisition, Investigation, Methodology, Project administration.
